# Literature in Plague Time

**Published:** 1889-03-02

**Authors:** 


### LITERATURE IN PLAGUE TIME.*

*(Concluded from page 324.)*

### III.

Medicus' prescriptions are too complicated to be given here, and the best that can be said of any of them is that they will not do much harm; but his hygiene is better than his therapeutics. In plague time, he says, more than ever is temperance in eating and drinking desirable, and moderation both in labour and exercise. He objects to bathing, as tending to open the pores of the skin, but recommends strict cleanliness of both rooms and clothing, and a fire with perfumes burning in it to aid ventilation. With regard to the patient's surroundings, he makes the general remark that "Cattes, dogges, swine, duckes, doves, hennes, or gese, are very unwholesome nere unto the place or mansion of dwellyng, or lye dedde in ditches nere the town;" in which opinion most people will agree with him. Indeed, though Dr. Tocrub seems to have been overfond of a promiscuous mingling of drugs in his prescriptions, and of course thought blood-letting necessary under almost all circumstances—with the added cruelty of not letting the patient sleep for six or eight hours after the operation—he shows a pretty sound appreciation of the laws of health. Yet he is represented as a charlatan, seeking only to get as much as he can out of Antonius before the latter dies, and scoffing at Crispine, his apothecary, who in a fit of remorse wishes to destroy all his rotten drugs. Here again the author's prejudices lead him into inconsistency. He wants to represent the doctor as a scoundrel, but in his anxiety to tell the public of the measures he advises for the prevention and cure of the epidemic, he is forced to make Tocrub appear as a benefactor by putting all these wise speeches in his mouth.

While the doctor and the apothecary hold the citadel of the sick-chamber, there come to the door "two Pettifoggers in the Lawe," Avarus and Ambodexter, anxious to draw up a will for Antonius. It is needless to say that they are rascals; was there ever a lawyer in fiction who was not! But on this occasion the doctor outwits them. He give such an account of the patients condition that they postpone their intentions till another day, and depart without seeing him; consoling themselves, however, by stealing the doctor's mule.

It is rather a relief to get away from this amiable gathering to join Civis and his wife again. They have determined at last to leave the infected town, and set out on horseback, with Madame riding on a pillion behind Monsieur, attended by Roger, their servant, who possesses "the gift of the gab." He has comments to offer on all they see, stories to tell *apropos* of every word they utter—sometimes an anecdote about his grandfather's experiences in the Wars of the Roses, or again a fable from the great story-book of the Middle Ages, "Reinecke Fuchs." Uxor makes one or two attempts to keep the groom in his place, but Civis himself rather encourages the conversation. On the whole, we agree with Civis, for Roger's chat is amusing, though we can understand that his mistress is not greatly taken with it when he explains that the reason dogs no longer speak articulate language, as they used to do in the days of myth and fable, is "that there are so many languages nowadays that they cannot tell which to speake—so leave all alone, and tourneth all to plaine barkynge—as women do as when they do fall from reasoning into scolding"

But death is not easy to escape, and Civis, flying from the town to avoid it, meets a traveller called "Mors" on the country road. Death's description of himself is marked by a gloomy power that is not to be found in any other part of the dialogue. He speaks like the chorus of a tragic drama, with a terrible force not unworthy to be compared with Raleigh's "O eloquent, just, and mighty death . . . thou hast taken all the pride and glory of this world, and covered them with these two narrow words, '*Hic jacet!*'"

With Civis' death the dialogue comes to an end. We learn incidentally from Theologus, who comes to comfort his last moments, that Antonius is dead, and Avarus and Ambodexter are preparing his funeral. The dialogue ends sadly enough. Yet not altogether sadly, for the last word is left to Theologus, who speaks of redemption, consecration, and eternal glory. This story of disease and death does not end with the grave, but strikes the higher note of resurrection.

* "A Dialogue Against the Fever Pestilence." By William Bullein. From the edition of 1578, collated with the earlier editions of 1564 and 1573. Edited by Mark W. Bullein and A. H. Bullein. London: Published for the Early English Text Society by N. Trübner and Co., 57 and 59, Ludgate Hill. Price Ten Shillings.

